# The immunology of B-1 cells: from development to aging

**DOI:** 10.1186/s12979-024-00455-y

**Published:** 2024-08-02

**Authors:** Matheus Silvério Mattos, Sofie Vandendriessche, Ari Waisman, Pedro Elias Marques

**Affiliations:** 1 Laboratory of Molecular Immunology, Department of Microbiology, Immunology and Transplantation, Rega Institute for Medical Research, KU Leuven, 3000 Louvain, Belgium; 2 Institute for Molecular Medicine, University Medical Centre of the Johannes Gutenberg University of Mainz, Mainz, Germany

**Keywords:** B-1 cell, Natural antibody, Aging, B cell development, Immunology, Autoantibodies

## Abstract

B-1 cells have intricate biology, with distinct function, phenotype and developmental origin from conventional B cells. They generate a B cell receptor with conserved germline characteristics and biased V(D)J recombination, allowing this innate-like lymphocyte to spontaneously produce self-reactive natural antibodies (NAbs) and become activated by immune stimuli in a T cell-independent manner. NAbs were suggested as “rheostats” for the chronic diseases in advanced age. In fact, age-dependent loss of function of NAbs has been associated with clinically-relevant diseases in the elderly, such as atherosclerosis and neurodegenerative disorders. Here, we analyzed comprehensively the ontogeny, phenotypic characteristics, functional properties and emerging roles of B-1 cells and NAbs in health and disease. Additionally, after navigating through the complexities of B-1 cell biology from development to aging, therapeutic opportunities in the field are discussed.

## Highlights

• B-1 cell development is regulated by specific transcription factors and this innate-like B cell bypasses selection phases during lymphopoiesis to generate polyreactive BCRs.

• B-1 cell-derived cytokines have immunoregulatory effects during homeostasis and diseases.

• Natural antibodies act as “eat-me” signals for the phagocytosis of cellular debris and invading pathogens.

• Impairment in natural antibody function is correlated with onset and progression of chronic diseases in the elderly.

## Significance

Recent research has brought the spotlight back to B-1 cells and natural antibodies, highlighting them as promising therapeutic targets. Natural antibodies are involved in homeostatic functions such as clearance of cellular debris and their presence is correlated with reduced chronic diseases in the elderly. Additionally, development of vaccines that generate long-term production of these broadly neutralizing immunoglobulins could be the future in tackling pathogens that are rapidly mutating, such as viruses. Understanding the immunology of B-1 cells can provide new insights for their use in clinical practice.

### B-1 cell: a unique innate-like lymphocyte

The B cell compartment is recognized by its clonally diverse population, bearing receptors that confer to these lymphocytes the ability to anticipate any antigen they will encounter during an organism’s lifetime. The diversity in lymphocyte receptors is generated somatically, mostly through combinatorial mechanisms during gene rearrangements that take place in B cell lymphopoiesis. There is, however, a subtype of B cell that generates a B cell receptor (BCR) that conserves its germline characteristics and has biased V(D)J recombination. These lymphocytes are termed B-1 cells to differentiate them from the well-known “conventional” B-2 cells Table 1. B-2 cells are the predominant subtype of B cells in adults and can be easily found in secondary lymphoid organs where they form germinal centers upon T cell-dependent activation [1]. B-2 cells are responsible to generate adaptive high-affinity antibodies after increasing their junctional diversity through somatic hypermutation of the BCR. Unlike their conventional counterparts, B-1 cells are mostly present in peritoneal and pleural cavities where they can spontaneously differentiate into plasma cells in a T cell-independent manner, and produce natural antibodies (NAbs) with polyreactivity, reduced junctional diversity, less somatic hypermutation and lower affinity [2]. As this review is focused on B-1 cells, we use the term “natural antibodies” to refer to any antibody that is produced by B-1 or B-1 derived plasm cells. This innate-like feature endows B-1 cells with the ability to respond rapidly to invading pathogens, positioning them as essential components of the innate immune system.

**Table 1:** Defining B-1 cells

In the early 1980s, it was observed that some cancerous B cells from patients with chronic lymphocytic leukemia expressed the T-cell marker CD5 (formerly known as Ly-1), which was later also found in mouse B cell lymphomas [3]. These findings instigated the search for non-cancerous B cells expressing CD5, which were found in low frequency in the spleen but in high frequency within the peritoneal and pleural cavities of healthy mice [4]. Murine B-1 cells are now phenotypically characterized by their surface markers IgM^high^ IgD^low^ CD19^high^ B220^low^ CD23^−^ CD43^+^ with CD5 distinguishing between B-1a (CD5^+^) and B-1b (CD5^−^), while the follicular B-2 cell is characterized by CD19^+^ B220^+^ CD23^+^ and CD43^−^. B-1 cells also express the integrin CD11b when inside serous cavities, but this marker is lost upon their migration to lymphatic vessels or spleen [5]. Besides the difference in CD5 expression, it was proposed that there is a division of labor between B-1a and B-1b cells [6], although this is still controversial. Additionally, it was recently shown that B-1a cells down-regulate CD5 expression after Toll-like receptor (TLR) stimulation [7], suggesting that B-1b cells might be only the activated form of B-1a cell. Although these surface markers constitute an easy way to differentiate B-1 from B-2 cells, many of these markers either do not encompass all B-1 cells or they can sometimes also be expressed by B-2 cells. Currently, the best way to distinguish B-1 cells and their antibodies from B-2 cells is the neonatal chimera model, in which host B-1 cells are replaced in neonatal mice by a congenic Ig-allotype-disparate donor B-1 cells while the B-2 cells remain of the host [8]
Whereas murine B-1 cells are well-characterized, the phenotypic markers for human B-1 cells are still not fully established. It has been proposed that circulating B-1 cells in humans are characterized by the surface markers CD19^+^ CD20^+^ CD27^+^ CD38^low/int^ CD43^+^. Further analysis demonstrated that 75% of cells bearing this phenotype express CD5 and share homologous functions with their murine counterpart, such as spontaneous antibody secretion [9]. Considering that CD43 can also be expressed by activated B-2 cells, the authors confirmed that these cells did not express other characteristic markers for B-2 cell activation such as CD69 and CD70 [9]. Thus, some characteristics that are ascribed to human memory B cells (identified by CD27 expression) are actually specific of B-1 cells [9]. Using spatial transcriptomics, a more extensive characterization of human B-1 cell was provided by identifying prenatal B-1 cells expressing CD5, CD27 and CD43 [10]. The authors also identified a subset of B-1 cells that express high levels of CCR10. These CCR10^+^ B-1 cells are highly proliferative and have shorter N-additions in the complementarity-determining region (CDR)3 junction in both immunoglobulin (Ig) heavy and light chains. They also confirmed that B-1 cells have the capacity to spontaneously secrete antibodies. The proportion of B-1 cells decrease from the first to the second trimester of gestation in almost every organ, except for the thymus, where the population of B-1 cells persisted [10]. Human B-1 cells and its ontogeny was recently reviewed by Kageyama *et. al.,* [11]

B-1 cells were initially overlooked because they are self-reactive, thus, going against Burnet’s clonal selection theory. In the last decades, B-1 cells have regained the attention of immunologists as key players in keeping tissue homeostasis and NAbs are now recognized by their essential role in the clearance of dead cells in tissues. Nevertheless, despite the significant progress in understanding B-1 cell biology, several questions persist. In addition to their canonical role in producing NAbs, B-1 cells have part in immunoregulatory processes [12] and in the generation of immunological memory [13]. Its physiological relevance is highlighted by the fact that NAbs are evolutionarily conserved and were found in every Gnathostomata (jawed vertebrates) species in which it was investigated, from fish [14] to mammals [15]. In this review, we discuss the complexity of B-1 cell biology and their specialized role in the immune system, while providing a comprehensive overview of their functional properties and emerging roles of NAbs in health and disease.

### Molecular cues and transcription factors in B-1 cell lineage commitment

**Fig. 1:**
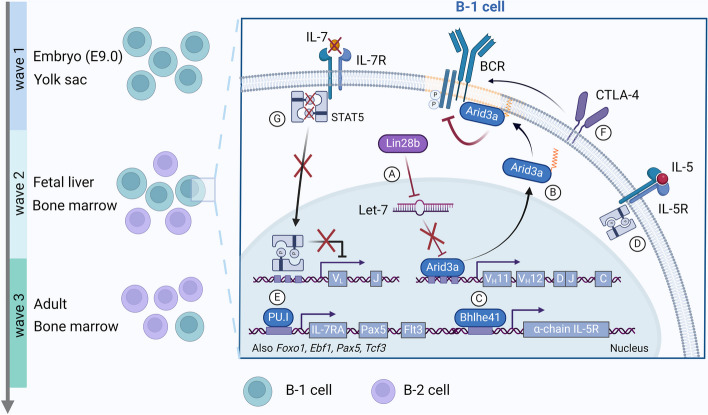
Key molecular events in B-1 cell development. B cell lymphopoiesis takes place in three waves. The first wave starts at E9.0 at the yolk sac. This wave is HSCs-independent and generates only B-1 cells. The second wave takes place in the FL, it is HSCs-dependent and can generate mainly B-1 but also B-2 cells while the third wave happens in the adult BM and mainly generates B-2 cells. (**A**) Lin28b is responsible to downregulate the micro–RNA Let-7 which normally prevents the activation of the transcriptional factor Arid3a. Activated Arid3a can migrate to the nucleus and bias the choice of V_H_ during VDJ recombination. (**B**) Additionally, Arid3a can be palmitoylated in the cytoplasm and associate with BCR-containing lipid rafts, increasing the threshold required for BCR activation. (**C**) Bhlhe41 regulates the expression of the α-chain of the IL-5 receptor. (**D**) IL-5R signaling is required for B-1 cell proliferation and self-renewal. (**E**) PU.1 controls the expression of key genes in B-1 cell development such as *IL-7Ra*, *Pax5* and *Flt3*. (**F**) CTLA4 is essential for the stability of the BCR and to control B-1 cell proliferation. (**G**) Absence of IL-7 signaling prevents pSTAT5-mediated inhibition of Ig_K_ recombination in FL

B-1 cell development is still a controversial topic (Table 2). The multi-layered origin of B1 and B2 cells is currently proposed to happen in 3 waves of B-1 and two waves of B-2 cell development. In mice, the first wave is independent of hematopoietic stem cells (HSCs), occurring at embryonic day (E)9 in the yolk sac (YS) [16], and generate only B-1 cells [17]. The second wave takes place in the fetal phase, where HSCs in the fetal liver (FL) can give rise to B-1 and B-2 cells, while the third wave occurs in the adult bone marrow and primarily generates B-2 cells [17] (Fig. 1). It was recently demonstrated that peritoneal B-1a cells are derived from the first wave and that fetal and neonatal HSCs did not contribute to the pool of peritoneal B-1a cells [18]. This divergent developmental program is inexorably associated with transcription factors and molecules that will guide this process differentially during fetal and adult phases.

**Table 2:** B-1 cell ontogeny: made or born?

There are two theories explaining B-1 cell origin. The *selection model* postulates that B cell progenitors are instructed to become either B-1 or B-2 based on the BCR-mediated recognition of antigens. The *lineage model* posits that B-1 and B-2 cells arise from different progenitors, which are committed with each particular lineage even before the expression of the BCR. Data supporting the *lineage model* arose from experiments showing that adult BM-derived HSCs can efficiently reconstitute B-2 [19] cells but they poorly reconstitute B-1a cells [20], whereas neonatal liver-derived cells can efficiently reconstitute the B-1 cell pool [21]. In 2006, Montecino-Rodriguez and colleagues described a specific B-1 cell precursor (Lin^−^CD93^+^CD19^+^B220^lo/neg^) that was found in the fetal liver [22], which was later found in the yolk sac (YS) at day E9.5 [16]. This early hematopoietic progenitor at E9.5 preferentially generated B-1 rather than B-2 or marginal zone B cells [23]. Considering that HSCs capable to generate B-2 cells appear only at E10.5 in the AGM region, B-1 cells can be generated in a pre-HSC wave of lymphopoiesis [16]
The selection model arose after a publication by Cong and colleagues showing that splenic B cells, lacking CD5 and expressing CD23, assumed the B-1 phenotype expressing CD5 after culturing in the presence of anti-IgM and IL-6 [24]. Subsequently, Haughton et al., proposed that “B-1 cells are made, not born”, starting the hypothesis that the commitment to B-1 or B-2 lineage occurs after BCR expression and is antigen-driven [25]. In fact, the generation of CD5^+^ B-1 cells is strongly dependent on BCR signaling, since Btk-deficient Xid mice have fewer B cells in the spleen, low levels of serum IgM and completely lack CD5^+^ B-1 cells [26]. More direct evidence for the role of BCR signaling in the instruction of B-1a cells was provided by experiments using transgenic mice that naturally generated self-reactive B cells expressing a BCR that recognizes the cell-surface protein Thy-1 (CD90). While B-1a cells in mice expressing Thy-1 were efficiently generated, mice deficient for Thy-1 could not generate B-1a cells [27]. In addition, the swapping of specific BCRs in B-2 cells is sufficient to switch B-2 cells into B-1 cells in transgenic mice. The switch induces proliferative burst and the migration of these cells to the peritoneal and pleural cavities [28]

Differential expression of *Lin28b* and *Let-7* genes was observed in cells purified from FL and it was proposed to promote fetal development of B-1a cells [29]. Lin28b is an evolutionarily conserved protein that downregulates the translation and maturation of the micro (mi)RNA let-7 [30]. The transcription factor Arid3a is targeted by the miRNA Let-7, a process that is key in B-1 cell development [31]. It was proposed that Arid3a could preferentially bind to some variable genes of the heavy chain (V_H_) during VDJ rearrangement, biasing the Ig heavy-chain (IgH) expression and selecting for those BCRs normally expressed by B-1a cells [31] (Fig. 1A). Additionally, Arid3a can be palmitoylated in the cytoplasm and alter BCR signaling due to its association with BCR-containing lipid rafts [32] (Fig. 1B). Higher levels of Arid3a decrease BCR signaling during B cell development, facilitating the selection of autoreactive BCRs [31]. Definitive FL-derived HSCs at E14.5, in mice, are sources for both B-1a and B-2 lineage precursors. However, all bipotent FL-derived HSCs become restricted to originate only B-2 cells over time. This attenuation of the B-1a potential is associated with the loss of *Lin28b* expression [33].

Another transcription factor involved in B-1a cell development is the basic helix-loop-helix family member e41 (Bhlhe41), which is expressed in B-1a transitional cells in the neonatal spleen [34]. B-1a cells deficient in Bhlhe41 exhibit altered cell surface markers and BCR repertoire with the loss of their prototypical phosphatidylcholine (PtC)-binding V_H_12/V_K_4 BCR [34]. Bhlhe41 and Bhlhe40 are key in controlling B-1a cell proliferation and survival, since both are required for the expression of the α-chain of the IL-5 receptor [34] (Fig. 1C), whose signaling is implicated in the self-renewal of B-1a cells [35] (Fig. 1D). PU.1 is another key transcription factor in B-1 cell development, acting upstream of key B-cell-specification factors such as Pax5 and the receptors Flt3 and IL-7R. Because of this, PU.1 was initially believed to be critical for the development of all B cell subsets, however, deletion of the gene *Sfpi1* (encoding PU.1) did not affect fetal B cell lymphopoiesis and induced a B-2 to B-1 cell switch [36]. The expression of the transcription factors *Foxo1, Ebf1, Pax5* and *Tcf3(E2A)* are higher in B-1 than in B-2 cells at all developmental stages [17]. The absence of pre-HSC B-1 wave in PU.1 deficient-mice correlates with the absence of IL-7Rα-expressing progenitors (IL-7 signaling is not required for fetal B-1 cell development, see below), which is consistent with works showing that PU.1 regulates the expression of this receptor [37] (Fig. 1E).

The cytotoxic T lymphocyte antigen 4 (CTLA-4), a surface molecule expressed by conventional and regulatory T cells, is also key in regulating B-1a cell development. CTLA-4-deficient B-1a cells have proliferative burst, internalize their BCR (Fig. 1F) and the resultant IgM^−^ B-1a cell, shifts to an antigen presenting cell (APC) phenotype (CD95^+^ CD38^lo^ GL7^+^ PNA^+^ CD150^+^ csIg^+^). These cells further upregulate MHC II and the chaperone H2-DM, which has central role in loading antigen in the MHCII. When these B-1a-derived APCs are transferred to IgH allotype-congenic recipients, they can activate T follicular helper cells and induce germinal center formation in recipients’ spleens [38].

B-1 cells acquire their unique characteristic by bypassing the pre-BCR selection stage (Table 3). In the FL, reduced IL-7R/STAT5 signaling induces early light chain recombination during the Pro-B cell stage (Fig. 1G). This is the same stage when the heavy chain VDJ recombination occurs. Thus, immature B cells in the FL can mount the mature BCR, dispensing the need of a surrogate light chain (SLC) to mount a pre-BCR during the large pre-B cell stage. During the large pre-B cell stage, BCR activation induces positive selection and cell proliferation instead of negative selection and cell death [39], and this may be what locks the B-1 cells in their characteristic proliferative burst. In mice expressing a constitutive active form of STAT5 (*Stat5b-CA*), B-2 and B-1b cell development are not affected while the development of B-1a cells is reduced [39]. Characteristic heavy chains expressed by B-1a cells such as V_H_11 pair poorly with SLC [40] and in mice lacking SLC, the development of B-1 cells is not affected [41]. At E9-11, murine lymphoid progenitors cells express *Rag2* and *VpreB* but lack the expression of immunoglobulin lambda-like polypeptide 1 gene (*Igll1*). *Igll1* encodes a protein that forms the SLC and its delayed expression means that there is a phase in mouse development in which B cell lymphopoiesis can occur in the absence of SLC.

**Table 3:** Pre-BCR selection and N-additions in B-2 cells

The development of B cells in the BM occurs in two main sequential steps of immunoglobulin gene rearrangement. VDJ recombination occurs first in the heavy-chain locus (Ig_H_) at the early pro-B cell stage by first recombining the heavy chain diversity (D) to the joining (J) gene segment. Later, the variable (V) to DJ segment is rearranged at the late pro-B cell stage. The resultant Ig_H_ pairs with a surrogate light chain (SLC) which is composed by VpreB and lambda5 proteins, forming the pre BCR. At this moment, successful signaling from the pre-BCR is required for the proliferative burst of the pre-B cell and subsequent rearrangement of the light chain (Ig_K_) V to J. The cytokine IL-7 is key in directing the sequential ordering of these recombination events. Through IL-7R and its downstream signaling STAT5, IL-7 actively inhibits *Igk* rearrangement [39] (Fig. 1G). In B-2 cells, the pre-BCR is an additional mechanism of negative selection for self-reactive Ig_H_ chains, since the SLC pairs poorly with autoreactive IgH [40]. During B-2 cell lymphopoiesis in the adult BM, any B cell that generates self-reactive BCR will undergo a process of negative selection which culminates in further V(D)J recombination or, ultimately, cell death by apoptosis [42]
V(D)J recombination requires DNA strand breaks. Before the DNA ends are rejoined, non-templated (N)-nucleotides are added by the enzyme terminal deoxynucleotidyl transferase (TdT) [43]. TdT is an unusual DNA polymerase that catalyzes the template-independent addition of random nucleotides being expressed in the pro-B cell stage. However, TdT is not expressed during the fetal stage in mice, thus, little to no N-addition is found in FL-derived B cells [44], which is the case of B-1 cells. In humans, TdT is expressed during the fetal phase, and both fetal and adult human B cells produce Ig with N-additions [45]. However, it has been demonstrated that the number of N-additions and CDR3-H3 length in B cells from preterm and term infants are shorter than those in adults [46]

### B-1 cell distribution and functional diversity

B-1 cells are particularly abundant in body cavities, but they are also present in the spleen, BM, mucosal tissues from gastrointestinal and respiratory tract, bloodstream, and less in lymph nodes [47]. The functional repertoire of B-1 cells is distinct according to their location; about 10% of B-1a cells in the peritoneal cavity recognize and bind to liposomes containing phosphatidyl choline, while only 2% of the B-1a cells in the spleen bind to these liposomes [48]. This functional diversity is mirrored by the V_H_ chain usage between splenic and peritoneal B-1 cells. For instance, splenic B-1a cells favors the usage of V_H_ V6-6 (J606), V11-2 (V_H_11) and V2-6–8 (Q52), whereas peritoneal B-1a cells are further enriched with V9-3 (Vgam3.8), V2-9(Q52) and V2-6–8 (Q52) genes [49].

**Fig. 2:**
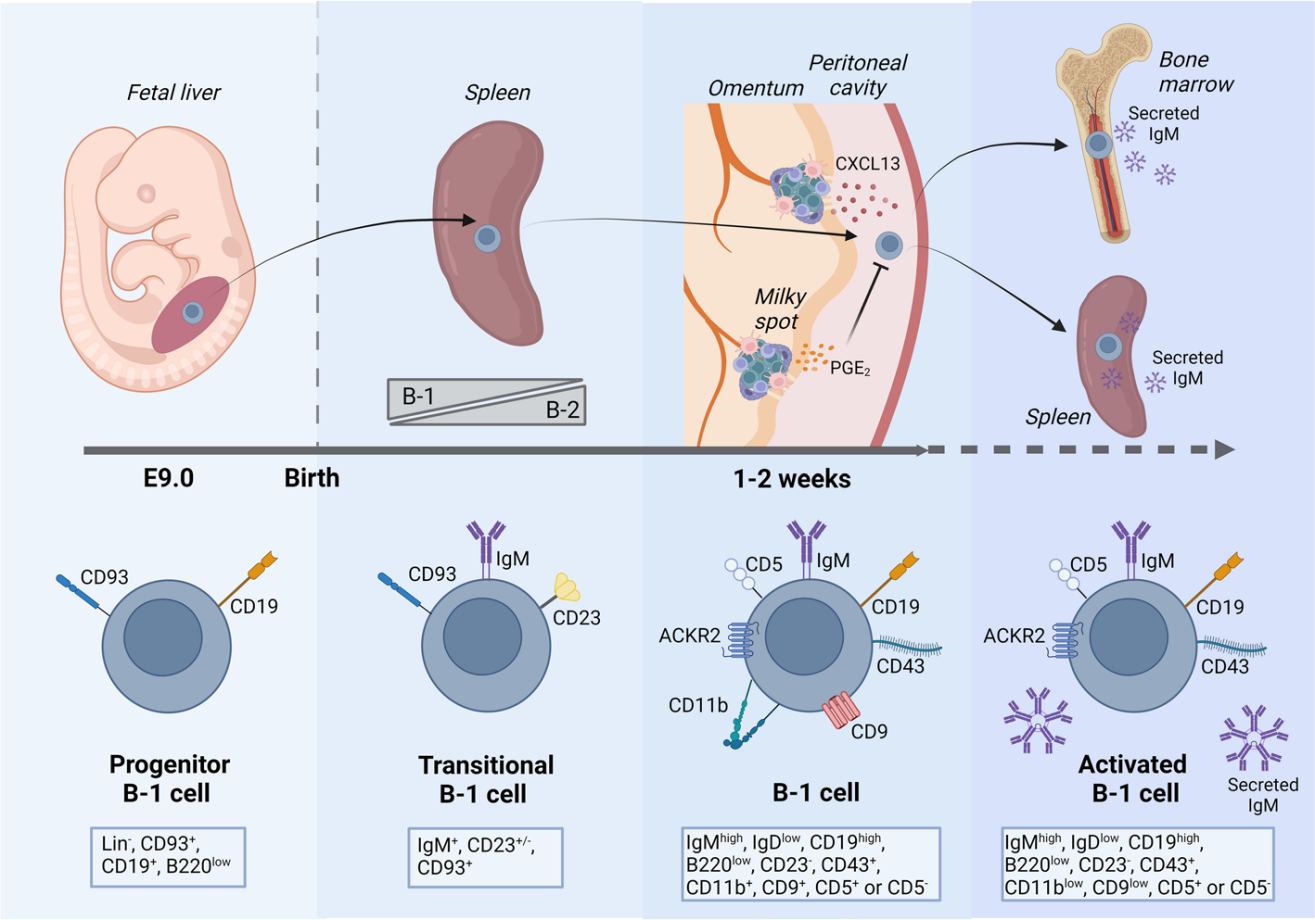
B-1 cell migration and tissue distribution. Progenitor B-1 cells (Lin^−^ CD93^+^ CD19^+^ B220^low^) migrate from FL to the neonate spleen where they can be found as transitional B-1 cells (IgM^+^ CD19^+^ CD23^±^ CD93^+^) until the second week of life. At this moment, transitional B-2 cells starts to arrive in the spleen and the transitional B-1 cells migrates to the body cavities where they arrive trough the omentum and can be found as mature B-1 cells (IgM^high^ IgD^low^ CD19^high^ B220^low^ CD23^−^ CD43^+^ CD11b^+^ CD5^+^ or CD5^−^). Upon activation, B-1 cells downregulate CD11b and CD9 and migrate to the spleen where they upregulate CD6 which is required for B-1 cell proliferation in this compartment. At spleen and in a less extent in the BM, B-1 cells spontaneously differentiate into plasma cells and secret natural IgM

Experiments in which B-1 cells were collected from the peritoneum of adult mice and transferred to newborn allotype-congenic mice showed that the pool of B-1 cells was completely restored in every niche, including peritoneum, spleen and BM [50]. This could be explained in two ways i) the body cavities act as a reservoir of B-1 cells, having a heterogenous population capable to replenish the whole pool of B-1 cells or ii) that B-1 cells are plastic and can be modulated by tissue-specific signals. The second hypothesis is more likely to be true considering the support in the literature [51]. A remarkable difference between B-1 cells in different compartments is that the production of NAbs is largely restricted to the B-1 cells in the spleen and BM [52]. The secretion of NAbs by B-1 cells in the peritoneum is inhibited by prostaglandin E2 released by activated peritoneal macrophages [53] (Fig. 2). Additionally, populations of bona-fide tissue-resident B-1a cells were identified in the lungs, liver, kidney and bladder [54]. These resident B-1a cells are spatially colocalized with resident macrophages and have a role in regulating their polarization through IL-10 production, promoting an anti-inflammatory phenotype and decreasing their ability to clear bacteria [54].

The exact mechanism by which B-1 cells arrive in body cavities is still not fully understood. B-1 cells start to accumulate in the peritoneal cavity 1–2 weeks after birth and this is dependent on the chemokine CXCL13 [55] (Fig. 2). In contrast to their precursors, mature B-1 cells are not present in the FL. Analysis of FL during E19 showed that IgM^+^ cells represent only 0.6% of the total CD19^+^ cells and only 20% of these IgM^+^ cells were expressing the markers of B-1a cells CD43^+^ CD5^+^ [49]. This suggests that B-1 cells start to migrate from FL to body cavities while still immature, potentially as a transitional B-1 cell. Indeed, transitional B-1 cells (CD93^+^ IgM^+^ CD23^±^) are found in the spleen during the first two weeks of life. Transitional B-1 cells then decrease in number in the spleen in the following weeks, while the B-2 transitional cells start to predominate in the adult spleen [56] (Fig. 2). This is also exactly when B-1 cells appear in body cavities, suggesting that these transitional B-1 cells are migrating from the spleen to body cavities. Transitional B-1 cells probably enter in the peritoneal cavity trough the omentum, which is an adipose tissue-derived from mesothelial cells located in the peritoneal cavity and connected to the stomach, spleen, pancreas and colon. Interestingly, the omentum of mice and humans contains “milky spots”, which are aggregates of leukocytes that resemble secondary lymphoid tissues [57] (Fig. 2).

The maintenance of B-1 cells in the body cavities is also dependent on the production of CXCL13. This chemokine is produced in milky spots, but also in similar structures called fat-associated lymphoid clusters which are found in the mediastinum, pericardium [58] and mesentery of mice [59]. Mice that lack secreted IgM contain few B-1 cells in the peritoneum while the B-1 cell population in the BM and spleen is not affected, indicating a role for secreted IgM in the accumulation of B-1 cells in the body cavities [60]. Although there is direct binding of IgM to B-1 cells, it is still unclear if this is due to a direct effect of IgM on B-1 cells or if this is due to lack of other “housekeeping” functions of natural antibodies, such as clearance of dead cells. Regardless of the mechanism, the fact that there are “normal” B-1 cell populations in BM and spleen while the peritoneal cells are reduced, suggests differential development between B-1 cells in different compartments. Interestingly, the migration of B-1 cells to the peritoneal cavity occurs only few weeks after the birth. Thus, if natural IgM is key for the development and maintenance of these cells in the body cavities, transitional or mature B-1 cells in the spleen might be the initial source of the natural IgM, which is consistent with previous work showing that there is a drastic reduction in peritoneal B-1 cells in splenectomized mice [47].

B-1 cells also express the atypical chemokine receptor 2 (ACKR2) which regulates their responsiveness to CXCL13 and the production of NAbs against PtC [61] (Fig. 2). B-1 cells express the integrin CD11b while in the body cavities and, together with CD9, it is a requirement for B-1 cells to adhere to the visceral and parietal mesothelium of the serous cavities. Upon activation of TLRs on B-1 cells, there is the consequent downregulation of CD11b and CD9 resulting in the detachment of B-1 cells from the matrix and their migration to other compartments [62] (Fig. 2). Activated B-1 cells migrate to the spleen or BM where they secret NAbs. B-1 cells that migrate to the spleen upregulate CD6, which is required for their proliferation and self-renewal in this organ, and CD6^−/−^ mice have impaired splenic B-1a cell proliferation [63] (Fig. 2). B-1 cells have increased rate of glycolysis, oxidative phosphorylation and they can internalize and store lipid droplets from the surrounding environment, all which further confirm how well these cells become adapted to where they reside. In fact, the lipid-rich environment in the peritoneal cavity is key for the self-renewal of B-1 cells, which is dependent on autophagy of the newly formed lipid droplets to provide the B-1 cells with fatty acids and energy [64].

### Natural antibodies: biased structure and polyreactivity

Nabs are circulating immunoglobulins mostly from IgM, IgG3 and IgA isotypes that arise early in life even in the absence of exogenous stimuli. NAbs are mainly produced by B-1 cells but not exclusively, since MZ B cells can also produce NAbs [65]. Although it was for long believed NAbs are produced in a T cell-independent manner, recent work showed that the development of B-1 plasma cells in the BM requires the presence of CD4 T cells, but it does not require antigen-specific interactions [66]. Initial evidence of NAbs production by B-1 cells originated from a study from Herzenberg’s group using autoimmune NBZ mice which spontaneously develop self-reactive antibodies and display high proportion of CD5^+^ B cells [67]. In sharp contrast to adaptive antibodies, which are antigen-specific, mono-reactive and recognizing mainly proteins, NAbs are polyreactive and recognize a myriad of antigens, including those of different molecular nature such as proteins, lipids, carbohydrates, nucleic acids and polysaccharides [68]. The term “natural antibody” was first introduced by Boyden in 1963 and since then it has been difficult to find a concrete definition for it. Considering that the antibody is the secreted form of the BCR, the best way to define NAbs is to take into consideration the molecular events that occur during gene rearrangement to generate the B-1 cell BCR and by the selection events following the BCR expression.

B-1 cells are biased in their choice of V_H_ and variable light (V_K_) genes to perform V(D)J rearrangement. For instance, the best characterized B-1 cells have a biased usage of V_H_11, V_H_12 and V_K_4 during gene rearrangement, thereby conferring to the resultant BCR the ability to bind to phosphatidylcholine (PtC) [69]. Moreover, the transgenic expression of V_H_12/V_k_4 BCRs results in the development of B-1 cells that produce NAbs able to recognize PtC [70]. Experiments using hybridomas from fetal and neonatal B-1a cells showed that these antibodies have few to no N-insertions (Table 3) and have biased usage of the proximal V_H_7183 and V_H_Q52 genes [71]. The prototypical B-1 cell-derived anti-phosphorylcholine NAb, T15, has also no N-addition. Thus, B-1 cells generate BCRs that conserve their germline-like characteristics.

### Polyreactivity

NAbs are recognized by their polyreactivity. It is important to highlight that polyreactivity is different from cross-reactivity, in which an antibody can bind to an off-target epitope but with high-affinity. To date, there is no definitive biochemical and/or biophysical model that explains what confers to an antibody the ability to bind with low affinity to multiple epitopes that are chemically and structurally diverse. It has been suggested that germline-like antibodies are more flexible in their complementarity-determining region (CDR) compared to high-affinity monoreactive antibodies due to the somatic hypermutation (SHM) at germinal centers for the latter, which incurs a substantial increase in stiffness of the CDR3-H3 loop [72]. As B-1 cells produce germline-like antibodies, NAbs remain then more flexible. However, research suggested that affinity maturation does not necessarily results in rigidification of the antibody [73], indicating that the mechanism is still uncertain. Moreover, the net charge of CDRs was suggested as a predictor of polyreactivity since antibodies with positively charged CDRs have the tendency to be polyreactive if compared with negatively charged CDRs [74].

The presence of arginine residues in the CDRs seems to be critical for polyreactivity and for the recognition of DNA and cardiolipin [74]. Increased prevalence of glycine, valine, tyrosine and tryptophan in polyreactive antibodies was also reported [75]. Every B cell tends to produce polyreactive BCRs during their early development, as pro-B cells, and this polyreactivity is reduced during the pre-BCR selection stage. The pre-BCR selection (Table 3) is also a mechanism that recognizes basic amino acids in the CDRs, such as arginine, and reduce their content in the mature BCR [76]. Therefore, the proportion of basic amino acids in pro-B cells is significantly higher than in pre-B cells [76]. This is further confirmed in SLC-deficient B cells, which lack pre-BCR selection, thus, the content of basic amino acids is similar in the mature BCR when compared to those in wild type pro-B cells [76]. Transgenic mice lacking SLC produce polyreactive IgG antibodies that bind to single-stranded DNA and polyreactive IgM that binds to single- and double-stranded DNA and to cardiolipin [76]. These antibodies were produced by CD43^−^ CD5^−^ B cells, meaning that they were not B-1 cell-derived NAbs [76]. Thus, bypassing the pre-BCR selection stage is also a feature that contributes to polyreactivity of NAbs.

### Class switch recombination

Most of the NAbs are IgM, but there are also IgG and IgA NAbs. Natural IgG is further subdivided into IgG1, IgG2, IgG3 and IgG4, with IgG3 being the most abundant [77]. The processes involved in B-1 cell class switch recombination (CSR) are not completely understood. CSR, just like SHM, is triggered by the enzyme activation-induced cytidine deaminase (AID) [78]. AID-deficient mice lack isotypes other than IgM and IgD in splenic B-1a cells [49]. Unlike the SHM and CSR in B-2 cells in germinal centers (which happen in a few days), SHM and CSR in B-1 cells start after weaning and increase progressively with age [49]. In this sense, the levels of class switched NAbs increase during 4–6 months after birth, mainly IgG3, IgG2b, IgG2c and IgA. Class switched NAbs have also a higher frequency of mutated sequences, suggesting that the increased CSR happens concomitant with SHM [49].

In contrast to natural IgM, the levels of natural IgA and IgG remain low in germ-free mice, suggesting that microbiota-derived molecules contribute to class switch in B-1 cells either through direct activation of TLRs in B-1 cells or by indirectly stimulating innate immunity [79]. Additionally, food-derived antigens might also influence the repertoire of IgG- and IgA-secreting cells [80]. In mucosal tissues, B cell activation is potentiated by TNF superfamily proteins BAFF [81] and APRIL [82] secreted by dendritic cells. These mediators induce the expression of AID and, in fact, the BAFF/APRIL pathway is key in the generation of IgG and IgA after microbiota colonization of the gut [83]. Administration of LPS into the peritoneal cavity promotes the migration of peritoneal B-1 cells to the milky spots [62], where they will differentiate into IgM- and IgA-secreting plasma cells, some of which will then colonize the intestine [84]. LPS administration also leads to the recruitment of GATA6^+^ macrophages to the omentum [85]. These macrophages express high levels of retinaldehyde dehydrogenase-2, an enzyme that catalyzes the generation of retinoic acid, a key factor in class switching to IgA [86].

### B-1 cell-derived cytokines

**Fig. 3:**
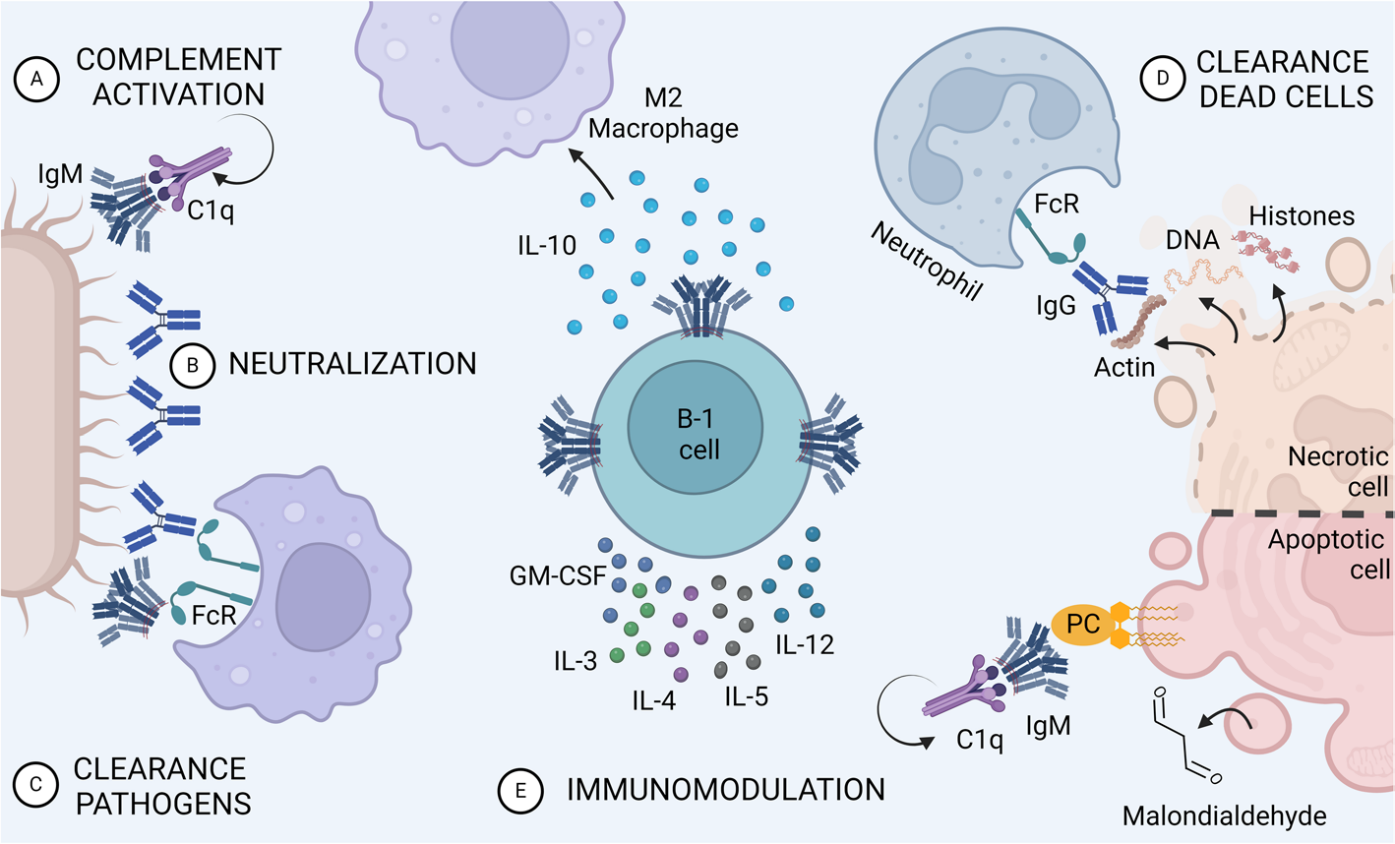
Homeostatic B-1 cell functions. B-1 cells secrete NAbs that act as first-line defense against invading pathogens and tissue damage by promoting: **A** complement cascade activation; **B** pathogen neutralization; **C** NAbs-dependent phagocytosis of invading pathogens; **D** opsonization and NAbs-dependent phagocytosis of dead cells; **E** modulation the immune system by secreting cytokines. PC: phosphorylcholine

B-1 cells can spontaneously secrete IL-10 and produce it in response to infections or sterile injury [87] (Fig. 3). Interestingly, pericardial adipose tissue is enriched in CXCL13, thus, it is rich in resident B-1a cells [88]. During myocardial infarct, the population of B-1a cells in pericardial adipose tissue increases and these cells start to release IL-10, improving the outcome of acute myocardial infarct by reducing inflammation, injury and preserving cardiac function [88]. B-1 cells have also an important role in secreting cytokines other than IL-10. It was demonstrated that B-1 cells secrete GM-CSF and IL-3 [89] during malaria infection. Interestingly, the initial source of these cytokines was IgM^+^ B-1b cells and then IgG^+^ plasmablasts, which may indicate a class switching in B-1b cells. B-1 cells also produce IL-4, IL-5 and IL-12 in response to *Propionibacterium acnes* polyssacharide [90]. Moreover, B-1 cells can secrete IL-27 containing exosomes that suppress and ameliorate uveitis [91] Thus, besides NAbs, B-1 cells are also relevant in the production of cytokines that can modulate immune responses triggered by infection or sterile stimuli.

## Natural antibodies in health and diseases

### NAbs in the clearance of dead cells

Apoptotic cell death is an integral part of the human development, necessary for proper growth and tissue remodeling. The role of NAbs in the clearance of apoptotic bodies is well described (Table 4, Fig. 3). In contrast, necrotic cell death culminates in plasma membrane rupture with the consequent deposition of intracellular contents in tissues. These necrotic debris will act as damage-associated molecular patters (DAMPs), triggering acute inflammation. While the recognition of apoptotic cells through phosphatidylserine (PS) is a very well-known process, the recognition of necrotic debris by phagocytes is still largely elusive.

**Table 4:** NAbs-dependent clearance of apoptotic cells

NAbs are well-known to bind to oxidation-associated neo-determinants that become exposed on the membrane of apoptotic cells. Phosphorylcholine (PC) is an example of neo-determinant present in the plasma membrane of apoptotic cells. It is a head group of neutral lipids such as phosphatidylcholine, but it is kept in a conformation that hides it from recognition by antibodies. When cells undergo apoptosis, oxidative alterations change lipid conformations, exposing PC [92]. Despite the variety of neo-epitopes exposed on the plasma membrane of apoptotic cells, around 50% of splenic B-1 cells recognize PC and malondialdehyde after immunization with apoptotic cells [93]. However, as B-1 cells rapidly divide and downregulate CD5 expression upon exposure to antigens, this percentage of B-1 cells binding to AC after immunization does not reflect the frequency of AC-binding B-1 cells in unimmunized mice. However, it highlights the ability of these cells to sense and react to common neoepitopes exposed upon apoptotic cell death.” In fact, the prototypical NAb, T15, is recognized by its ability to bind to PC-containing antigens [94]
Binding of natural IgM anti-PC to apoptotic cells leads to further opsonization with the eat-me signals C1q and MBL, subsequently triggering complement activation. [93]. Opsonization of apoptotic cells with NAbs also allows recognition by FcRs on professional phagocytes, which will ultimately result in phagocytosis of dead cells. Interestingly, natural IgM and IgG do not bind to PS [68], thus, NAbs may act as a complementary mechanism, together with PS, in the clearance of apoptotic cells. The role of natural IgM in the clearance of apoptotic cells is well stablished in vitro and in vivo by pioneering studies. Incubation of apoptotic bodies with polyclonal IgM increases their clearance [95]. Natural IgM was also demonstrated to increase the phagocytosis of apoptotic cells by alveolar macrophages [96]. Mice that cannot secret IgM have impaired clearance of apoptotic cells, making them susceptible to develop lupus and produce autoimmune IgG against nucleic acids [97]. NAbs are also required for the removal of senescent red blood cells (RBCs), which cannot undergo apoptosis. As RBCs lack nuclei and mitochondria, the death of RBCs is not associated with energy-consuming events including the cleavage of intracellular content and alterations in membrane composition during apoptotic death. Some NAbs bind to senescent RBCs by recognizing determinants that involve the whole PtC molecule instead of only recognizing the PC-containing antigens [98]

We have recently shown that NAbs bind to necrotic cell debris and act as “eat-me” signals for the recognition and phagocytosis of necrotic cells [68]. Natural IgM and IgG were visualized recognizing and opsonizing the dying cells even before complete necrosis. In addition to oxidized lipids in the necrotic cell membrane, NAbs bound to intracellular components such as actin, DNA, cardiolipin and phosphoinositides, all of which are exclusive to intracellular compartments in healthy cells. Interestingly, no reactivity of NAbs was observed towards PS, the prototypical apoptotic “eat-me” signal. Opsonization with IgM and IgG NAbs was required for efficient clearance of necrotic cell debris by neutrophils and monocytes at sites of injury. IgG-debris immunocomplexes were recognized by FcγRs, mainly FcγRI, while IgM-coated necrotic debris phagocytosis was dependent on the integrin CD11b in both mouse and human phagocytes. Importantly, NAbs-mediated phagocytosis of necrotic debris induced the expression of IL-10 in macrophages, thus, NAbs signal for nonphlogistic phagocytosis of cell debris [68]. Overall, NAbs were central to promote liver regeneration post injury and even enhanced liver recovery when supplemented to wild-type mice. The abundance, immediate availability, polyreactivity and ability to bind to evolutionarily conserved antigens makes NAbs ideal to clean up the heterogenous debris released upon necrosis (Fig. 3).

### Natural antibodies in ischemia–reperfusion injury (IRI)

IRI is characterized by cellular dysfunction and necrotic cell death following the restoration of blood flow to previously ischemic tissues. Thus, the ability of NAbs to induce nonphlogistic clearance of necrotic debris suggests that these immunoglobulins could be relevant in clinical conditions with an underlying necrotic component, such as IRI during organ transplantation. In a mouse model of intestinal IRI, the number of peritoneal cavity B-1a cells is reduced and B-1a cell treatment at the time of mesenteric reperfusion inhibited the intestinal injury, systemic inflammation and remote injury in the lungs. The authors suggested that preserving the B-1a cell pool could be a therapeutic option for intestinal IRI injury [99]. On the other hand, another group found that the depletion of peritoneal B-1 cells by intraperitoneal injection of distilled water before IRI reduced renal injury [100].

Activation of the complement cascade has been proposed to exacerbate IRI. In a mouse model of post transplantation cardiac IRI, natural IgM was shown to bind to the neoepitopes and activate the complement cascade, resulting in overt inflammation and injury [101]. The role of natural IgM was also investigated in liver IRI. Using monoclonal natural IgM that recognizes Annexin IV, the authors suggested that natural IgM has a role in initiating an inflammatory response important for hepatic IRI, including the activation of complement. However, they also observe that this monoclonal IgM contributed to liver regeneration following 70% partial hepatectomy [102]. Thus, the role of B-1 cells and NAbs in IRI is still unclear, as works have provided contradictory data.

### NAbs in infectious diseases

The role of B-1 cells and NAbs in preventing bacterial and fungal infections is well documented and is summarized in Table 5 and Fig. 3. The constitutive production of NAbs as well as B-1 cell localization in the pleural cavity endow them as first-line responders upon lung infection/injury. Infection of mice with influenza virus causes strong and highly localized activation of B-1 cells in the draining lymph nodes of the respiratory tract, where these cells become a major source of virus-neutralizing IgM [103]. The accumulation of B-1 cells in lymph nodes occurs via type I interferon-dependent CD11b activation, which mediate the crossing of B-1 cells through the endothelium [104]. B-1a cells that accumulate in lymph nodes do not incorporate BrdU, thus, these cells respond to influenza infection by relocation and natural IgM production rather than clonal expansion [103]. In mice, infection with H1N1 results in the recruitment of B-1a cells to the lungs, where these cells will differentiate in high-rate IgM producing cells in an IL-17A-dependent manner, which is associated with survival upon H1N1 infection [105].

**Table 5:** Role of NAbs in diseases

	Disease/Model		Function	**Ref.**
**Mice**	Heart Disease	Atherosclerosis	Increased phagocytic uptake of oxLDL	[106]
			Reduced inflammation and atherosclerotic lesions	[107–110]
	Neurodegenerative diseases	Parkinson disease	Reduced neurodegeneration; improved synapse;Reduced motor dysfunction	[111]
		Alzheimer’s disease	Reduced neurotoxic effect of amyloid plaques to cultured neurons from mice and rats	[112, 113]
			Increased phagocytic uptake of plaques	[114–116]
			Reduced amyloid plaque deposition	[117]
	Organ injury and regeneration	Acute liver injury	Increased clearance of necrotic cell debris	[68]
		IRI	Increased complement-induced injury	[100, 101, 118, 119]
			Less organ injury and systemic inflammation	[99]
		Hepatectomy	Increased liver regeneration after partial hepatectomy	[102]
	Infectious diseases	Influenza	Increased viral neutralization	[103]
		*S. Pneumoniae*	Increased GM-CSGF release by recruited B1 cells	[120]
		*Candida *albicans	Increased fungal neutralization	[121]
		*Aspergillus fumigatus*		[122]
		*Cryptococcus neoformans*		[123, 124]
		Influenza H1N1	Increased survival	[105]
		Sepsis	Less systemic inflammation and improved survival	[125, 126]
		*Acinetobacter baumannii*	Protection against infection	[127]
		Malaria	Increased production of IL-3 and GM-CSF	[89]
	Cancer	Melanoma, Breast cancer,Adenocarcinoma	Inhibition of tumor growth	[128]
		Colon cancer	Inhibition growth of melanoma cells	[129]
	Auto-immune diseases	Systemic lupus erythematosus	Reduced IgG-autoantibodies and autoimmune disease	[97]
			Protection against lupus nephritis and (+) survival	[130–132]
**Human**	Cardiovascular diseases	Atherosclerosis	Reduced pathological uptake of oxLDL	[133]
			Reduced atherosclerosis progression and LPC cytotoxicity	[134]
	Neurodegenerative disease	Parkinson’s disease	Prevention of α-synuclein aggregation *in vitro*	[135, 136]
		Alzheimer’s disease	Protective anti-Aβ antibodies in blood and CSF	[137, 138]
			Less plaque burden	[139]
			Lower cognitive decline	[140]
	Cancer	Non-small cell lung carcinoma	Eliminates NeuGcGM3-expressing tumor cells in vitro	[141]
			Lower anti- NeuGcGM3 responses in NSCLC patients	
		Epithelial cancer	Increased apoptosis of carcinoma cells in vitro	[142, 143]
		Gastric cancer	Increased tumor-specific apoptosis	[144]
	Auto-immune diseases	Systemic lupus erythematosus	Protection against lupus nephritis	[145]

Nabs are emerging as broadly neutralizing antibodies important for protection against rapidly evolving viruses. In vaccinated humans or naturally infected with influenza A virus (IAV), more than 80% of the antibodies against the stalk region of hemagglutinin (HA) were polyreactive [146]. These polyreactive antibodies recognized conserved sites on HA in different strains of IAV H1N1 [146]. The diversity in HA among these strains reflects more than 100 years of evolutionary history, highlighting the power of broadly neutralizing antibodies. Polyreactive antibodies against HA were generated mainly after the immunization or infection with a novel IAV strain for which the patients were not currently immune [146]. These antibodies have biased usage of V_H_1-69 gene, which encodes more hydrophobic CDR loops and have less SHM. Thus, the development of vaccines that induce long-term production of NAbs or activation of B-1 cells will be of paramount importance in tackling rapid mutating viruses with pandemic potential such as influenza and HIV viruses.

### Aging

There are time-dependent alterations in NAbs repertoire, causing these relevant homeostatic immunoglobulins to drift away from their characteristic germline-like structure. These alterations result in loss of function of NAbs in the elderly which favor accumulation of noxious molecules in tissues and leading to the development of chronic diseases. Age-dependent loss of function of NAbs has been associated with clinically-relevant diseases in the elderly, such as atherosclerosis [147] and neurodegenerative disorders [148]. Because of these associations, NAbs were suggested as “rheostats” for the susceptibility of chronic diseases in advanced age [149].

In neonates, the BCR repertoire of peritoneal B-1 cells comprises multiple clones. However, as mice age, the diversity of B-1 cell population decreases, becoming large groups of a few clones expressing a more restricted BCR (e.g. *Ighv12-3/Igkv4-91* and *Ighv11-2/Igkv-126*) [150]. This phenomenon is also observed in humans [151]. Additionally, aging is associated with alterations in the net charge of the CDR-H3 region of the antibodies. The BCR of peritoneal B-1a cells that recognizes PtC becomes more charged (reduced hydrophobicity) with age, while the B-1a cells that do not recognize PtC show the opposite, a reduction in the charge of CDR-H3 with age [152]. Interestingly, there is no alteration in the net charge of CDR-H3 in any splenic B-1a cells, highlighting that tissue-specific signals select and modify the repertoire of resident B-1 CD5^+^ cells [152]. The reduced functionality of B-1 cells in the elderly is qualitative rather than quantitative. In the first week after birth, the peritoneal cavity of a C57BL/6 mouse is composed mainly by B-1 cell precursors (63%), where B-1 cells represent 21% and B-2 cells 16% [150]. In young adults (10- 11 weeks old), 87% are B-1 cells while 13% are B-2. In the elderly (15 – 19 months), the proportion of B-1 cells remains the same as in young adults being 88% B-1 and 12% B-2 [150]. It is important to highlight that these proportions will likely vary depending on the mouse strain and sex used for the analysis.

The age-dependent alterations in the NAbs repertoire are influenced by biological sex in mice. NAbs from aged female mice retain their protective capacity against pneumococcal infection while NAbs from male mice do not [153]. These differences are not only due to the levels of circulating NAbs, but also to differences in the B-1 cell population. Estrogen affects B cell development [154], maturation [155] and is required for production of protective NAbs against *E. coli* infection [156]. Young female mice have more peritoneal and splenic CD5^+^ B-1 cells than age-matched males [157]. Serum IL-5 levels are increased in aged female mice compared to aged males and IL-5 is key in the maintenance of B-1 cell population [158]. Aged females also have elevated expression of *Hmga2*, a transcription factor important for stem cell self-renewal [159]. Interestingly, *Hmga2* expression is regulated by the Lin28b/Let-7 axis [160], which is key for the B-1 cell development.

**Fig. 4:**
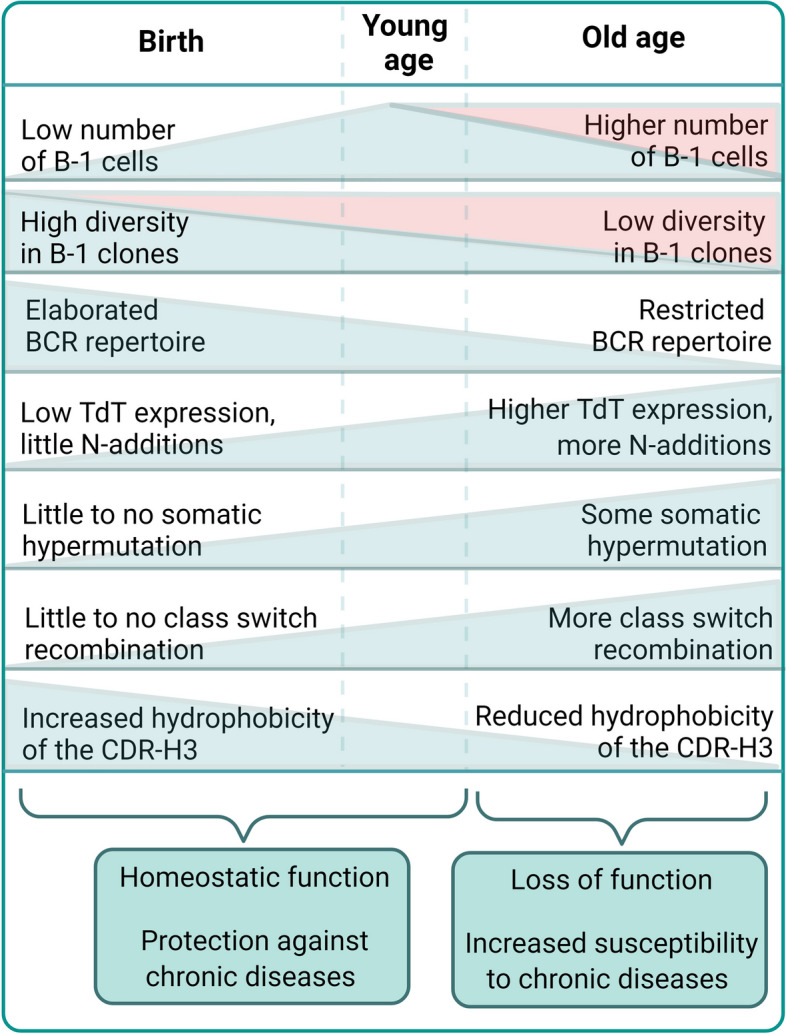
Age-related alterations in B-1 cells and NAbs. The number of B-1 cells in body cavities increases after birth until it peaks in the young adult and is kept until old age. However, tissue specific signals select B-1 cells expressing characteristic BCRs which culminate in the reduction in the diversity of B-1 cells. Although the B-1 cell population is maintained via self-renewal, B-1 cells can be generated de novo by adult BM-derived HSC, which now express TdT. This result in BCRs with more N-additions and more mutations. This, together with reduced hydrophobicity in the CDR-H3 loop, decreases the functionality of NAbs in the elderly, increasing the susceptibility to chronic diseases. The green shades indicate the general age-related alterations. The red shades indicate the age-related alterations in female mice

De novo generation of B-1 cells starts prior to birth and decreasing considerably in adults, meaning that B-1 cells will persist as a self-replenishing population. As discussed before, TdT expression is absent during the murine fetal development, initiating shortly after birth [44]. Around 60% of the IgH expressed by B-1a cells in the spleen from neonate mice between 2 to 6 days do not contain N-insertions in the CDR3, 30% contains 1–2, and less than 15% contains 3–4 N-nucleotide insertions [49]. After the first week of life, N additions starts to increase progressively until the 3rd week of life, which coincides with the weaning period. After this moment, the pattern of N-additions stabilizes, with 50% of CDR3 sequences containing 3–7 N nucleotides and 30% having more than 8 N-insertions. This pattern remains stable for at least the first 5 months of life [49]. The germline-like structure of NAbs in young mice (2 to 3 months old) is gradually lost by 6 months of age due to the increase in N-additions [161]. Alterations that push NAbs away from their germline-like structures (Fig. 4) are associated with loss of function, as showed by the loss of protection against pneumococcal infection. Although N-additions are found in human NAbs already in the initial phases of development due to the fetal expression of TdT, human and mouse B-1 cells contain few SHM [9], which increase over time and may lead to reduced function of NAbs in aged humans.

### Age-related changes in NAbs correlate with increased susceptibility to chronic diseases in the elderly

#### Atherosclerosis

Atherosclerosis develops due to accumulation of low-density lipoproteins (LDL) in the endothelium of arteries, where it becomes oxidized. Oxidized lipids are very reactive and can modify other molecules generating oxidation-specific epitopes (OSE) that will be recognized and phagocytosed by macrophages, leading to the formation of foam cells [162]. It has been reported since the late 1990’s that monoclonal IgM autoantibodies neutralize oxidized (ox)LDL, thereby inhibiting their pathological uptake by macrophages [106]. Also, increased circulating IgM levels decrease atherosclerotic lesion formation [107]. Deposition of IgM anti-oxLDL occurs in mouse and human atherosclerotic lesions [163], and administration or overexpression of anti-oxLDL antibodies reduced inflammation, lesion area and progression of atherosclerosis [108]. Natural IgM that binds to OSE on oxLDL such as anti-malondialdehyde (MDA-LDL) and anti-copper oxidized (Cuox)-LDL are considered to be atheroprotective [164]. Treatment of ApoE^−/−^ mice with natural polyclonal IgM reduced the levels of pathogenic CD4^+^ T cells in these mice [109]. B-1b cells are sufficient to produce IgM against oxLDL in vivo, providing atheroprotection in a mouse model of diet-induced atherosclerosis [165]. In LDL receptor-deficient mice, natural IgM is required for protection against atherosclerosis [166].

In a murine model of atherosclerosis, B-1a cells migrated from the peritoneal cavity to the BM in a CXCR4-dependent manner, where they secreted natural IgM against oxidation-specific epitopes [167]. This was also confirmed in a cohort of 50 patients suffering with atherosclerosis, in which it was demonstrated that the expression of CXCR4 is associated with high plasma levels of IgM anti malondialdehyde-LDL. They further observed that CXCR4 expression is inversely correlated with plaque burden and stenosis, but directly correlated with a more stable plaque phenotype [167]. The same group showed later that B-1 cells are recruited to the perivascular adipose tissue in a CCR6-CCL20 manner, where they will produce natural IgM [168].

#### Neurodegenerative disorders

Several neurodegenerative disorders are associated with deposition and formation of protein adducts in the brain. These aggregates are cytotoxic and must be removed to prevent neuronal death, increasing the severity of the disease. Two of the most burdensome neurodegenerative disorders are Parkinson’s disease (PD) and Alzheimer’s disease (AD), which are characterized by the presence of protein aggregates of α-synuclein or β amyloid (Aβ), respectively, in the brain.

Healthy humans produce NAbs that bind to these toxic proteins [169], but plasma and cerebrospinal fluid levels of NAbs against Aβ reduce with age and with the progression of AD. Natural IgG isolated from plasma of healthy controls protected primary cultured neurons from Aβ cytotoxicity [112]. Thus, in addition to their ability to increase the phagocytosis of these harmful proteins, NAbs can also reduce neurotoxicity by neutralizing them. Natural IgG levels are significantly reduced in brain regions severely affected by deposition of Aβ [170] and authors suggested that AD incidence might be the result of impairment in natural IgG-mediated Aβ clearance by microglia [170]. It was demonstrated that 17 NAbs have reduced plasma concentrations in AD patients and are correlated with loss of cognitive function [140]

Like Aβ, pathological increase in α-synuclein deposition is associated with increased inflammation in the brain. In the brain of PD patients, aggregates of α-synuclein are colocalized with IgG deposits [171]. However, the levels of NAbs against α-synuclein are reduced in PD patients compared to healthy controls [172]. Additionally, treatment with anti-α-synuclein antibody reduced neurodegeneration and rescued synapses [111], highlighting the protective role of antibodies against these proteins. A recent paper showed that natural IgG against Aβ is also reduced in patients with PD, suggesting that the ability to bind to Aβ and α-synuclein might be required to prevent the progression of PD [173]. The levels of anti- α-synuclein IgG2 are higher in PD patients compared to healthy individuals, but the levels of natural IgM and natural IgG4 are reduced [174]. Because of these neuroprotective effects, levels of circulating NAbs have recently being suggested as biomarkers for neurodegenerative disorders [175]. In fact, antibodies are stable molecules with long half-life and with fewer circadian cycle alterations, positioning them as great candidates to be used as biomarkers.

#### NAbs in cancer

Natural IgM has also been linked to protection against cancerous cells since it binds to tumor-specific glycolipids and carbohydrate structures. NAbs recognize some gangliosides (sialic acid-containing glycosphingolipids) that are found in the plasma membrane of essentially all vertebrate cells. These gangliosides undergo significant changes during malignant cell transformation. For instance, Neu5GcGM3 is a ganglioside that is absent in healthy human tissues but becomes present in several human tumors [176]. Interestingly, healthy humans produce natural antibodies anti-Neu5GcGM3, which recognize and eliminate tumor cells expressing this antigen. Patients suffering with non-small cell lung cancer have very low levels of anti-Neu5GcGM3 and B-1 cells are the main contributors for the natural IgM pool against Neu5GcGM3 [177]. B-1 cell-derived natural IgM was also reported as being protective in mouse models of peritoneal carcinomatosis [178] and colon cancer [129]. Moreover, NAbs were required for the elimination of precancerous cells in mice [128]. It was also proposed that activation of B-1 cells by administration of monophosphoryl lipid A and trehalose-6,6’-dicorynomycolate (TLR and C type lectin receptor agonists, respectively) effectively inhibited tumor growth and ascites in peritoneal carcinomatosis [178]. Thus, the presence of NAbs in the circulation is inversely correlated with the development and growth of tumors.

#### Autoimmunity and antigen presentation

The molecules recognized by NAbs encompass common self-antigens targeted by antibodies during autoimmune diseases, thus, it is reasonable to assume that NAbs might have a pathological role in autoimmune diseases. Although the exact role of NAbs in autoimmune diseases is not fully understood, research has shown that the presence of NAbs is correlated with prevention of autoimmune diseases. For example, mice that do not secrete IgM are defective in the clearance of apoptotic cells and develop autoimmune diseases [97]. On the other hand, treatment with IgM anti dsDNA resulted in a dramatic improvement in lupus nephritis and increased mouse survival [132].

The protective role of natural IgM in the development of autoimmune diseases was also shown in humans. The level of IgM against dsDNA is associated with protection in SLE patients for the development of lupus nephritis [145] and the IgG/IgM ratio of anti-dsDNA antibodies was proposed as a prognostic marker for lupus nephritis [179]. Treatment with recombinant self-reactive natural IgM reduced the levels of proinflammatory T_H_17 cells and controlled severity of lupus in FcγRIIB/TLR9 deficient mice [180]. Additionally, treatment with monoclonal IgM anti-dsDNA delayed the onset of lupus in (NBZ x NZW)F(1) prone mice [181].

#### Therapeutic opportunities and concluding remarks

NAbs have clearly beneficial roles in preventing the onset and progression of several diseases and the impairment in NAbs function in the elderly is associated with chronic diseases, autoimmunity and cancer. Thus, therapies that rescue the neonatal phenotype of B-1 cells, rescuing the diversity and germline-like structure of NAbs, could prevent the development of these diseases in aged patients. Intravenous immunoglobulin (IVIG) preparations have been largely used in clinical practice to treat several conditions. Considering that IVIG contains also NAbs, the identification of the beneficial NAbs in these formulations would have tremendous therapeutic value.

Nowadays, a major conundrum in generating successful vaccines against viruses is the current inability to induce broadly reactive antibodies after immunization. It is believed that polyreactive antibodies could recognize epitopes in pathogens that are normally rapidly mutating or that are shielded by glycosylation. Approximately 70% of broadly neutralizing HIV-binding antibodies are polyreactive, which is also observed in antibodies capable to bind to the hemagglutinin stalk domain in influenza viruses [182]. In fact, some HIV-1 neutralizing antibodies that were tested were able to bind to cardiolipin, PtC and PE, which may suggest that these are NAbs [183]. Polyreactivity is also suggested as a critical feature that allows the antibody to bind to imperfectly conserved epitopes on novel virus subtypes with pandemic potential. Interestingly, polyreactive antibodies seems to bind with increased affinity to HIV and influenza viruses if compared to monoreactive antibodies [184]. Thus, targeting B-1 cells during vaccination to generate a long-term production of NAbs could be a clinically relevant approach to tackle viral diseases in the future.

**Table:** 

Outstanding questions
• When during B cell lymphopoiesis do the B-1 and B-2 cell lineages diverge? It has been shown that CLPs can either generate B-1 or B-2, but never both, suggesting that the commitment to become each happens before the CLP stage • Why HSCs in the adult BM are skewed to generate B-1b instead of B-1a cells when transferred to lethally irradiated mice? • Do these different waves of B-1 cell development generate B-1 cells with different functionality and/or tissue distribution? • Could natural IgM against oxidized lipids in body cavities be responsible to retain the B-1 cells in the peritoneum in a Fcμ or CD11b-dependent way? • How can the rearrangement of different V(D)J genes result in similar pattern (frequencies) of polyreactive antibodies? • Could natural IgG from the mother cross the placenta and drive the initial B-1 cell development? • What is the homeostatic mechanism that keeps the total serum levels of NAbs? • Is there a role for natural IgA in mother’s milk in shaping the neonate microbiota? • Why does the N addition pattern stabilize after weaning? Is it due to antigens present in the solid food or due to the loss of maternal natural IgG from milk?

## Data Availability

No datasets were generated or analysed during the current study.
